# Gut microbiota and infant distress – the association between compositional development of the gut microbiota and fussing and crying in early infancy

**DOI:** 10.3402/mehd.v23i0.18577

**Published:** 2012-06-18

**Authors:** Anna Pärtty, Erika Isolauri

**Affiliations:** 1 Department of Paediatrics, Turku University Hospital, Turku, Finland; 2 University of Turku, Turku, Finland

**Keywords:** *infant crying*, *fussing*, *gut microbiota*, Bifidobacterium, Lactobacillus, *diary*

Excessive crying in an otherwise healthy child coincides with several environmental alterations and maturational processes: changes in the sleep and feeding patterns, immunological, endocrinological, and neurological maturation, thermoregulation, compositional development of the gut microbiota, and improvement of the immunological defences, including the gut barrier functions. An intimate interrelationship between diet, the immune system, and microbiome has been recognized when explaining susceptibility to disease later in life, subsequent to the demonstration that the establishment of the gut microbiota provides an initial and massive source of microbial stimuli for the maturation of the gut-associated lymphoid tissue, particularly for the IgA plasma cells, conferring the first line of host immunological defence. Notwithstanding the extensive and multidisciplinary scientific interest centred on infant nutrition and gut microbiota, research so far has been unable to conclusively ascertain the determinants underlying infant crying, a common problem manifesting itself at the peak of these maturational processes; the duration of crying increases after birth, reaching a maximum during the second and the third month of life (1). Infantile colic is a specific condition characterized by paroxysmal, excessive, and inconsolable crying, with a duration exceeding 3 hours a day for 3 days or more a week (2).

## Introduction

The attempts to control excessive crying, above crying related to acute infectious diseases, have focussed on varied dietary regimens of the affected child. Indeed, significant lessening in crying has been achieved by reducing the allergens: a subgroup of infants with excessive early fussing and colic-type cry manifests atopic diseases, or a heightened risk thereof, and food allergy (3–5). A potential role of specific probiotics is also suggested, as aberrant composition of gut microbiota has been reported in colicky infants during the time of colic (6–8).

## Aim

The aim of the present study was to establish whether there is an association between the compositional development of the gut microbiota and amount of fussing and crying in early infancy. We focussed on the entire spectrum of crying as opposed to the most often assessed colic cry (9).

## Methods

Behavioural patterns of 88 infants during the 7th and 12th week of life were recorded by parental diaries. During the first 6 months of life, infants’ gut microbiota was analysed firstly by quantitative polymerase chain reaction (qPCR) and fluorescent in situ hybridization (FISH) assays and secondly by PCR-denaturing gradient gel electrophoresis (PCR-DGGE). The study was approved by the Ethics Committee of the South-Western Finland Hospital District (9).

## Results and discussion

**Fig. 1:**
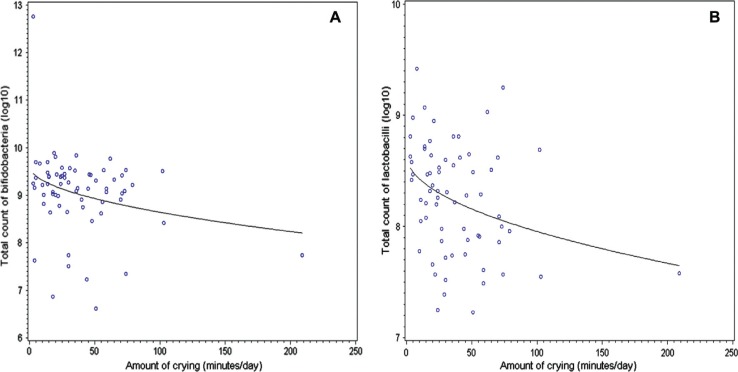
The total amount of crying (min/day) at the age of 3 months and the total count of *Bifidobacterium* (**A**) and *Lactobacillus* (**B**) at the age of 6 months.

The median (range) duration of total distress of the infants was 106 (0–478) min a day during the seventh week and 58 (0–448) min a day during the 12th week. At the age of 3 months, the proportion of *Bifidobacterium* counts to total bacterial count was inversely associated with the amount of crying and fussing during the first 3 months of life (*p* = 0.03). In contrast, *Bifidobacterium breve* behaved contrary to this general *Bifidobacterium* pattern: the amount of *Bifidobacterium breve* was found to be associated with the amount of total distress (*p* = 0.02). Furthermore, the prevalence of *Lactobacillus* spp. at the age of 3 weeks was inversely associated with total infant distress during the seventh week of life (*p* = 0.02). At the age of 6 months, after the peak period of crying, the total number of *Bifidobacterium* (*p* = 0.03) and *Lactobacillus* (*p* = 0.008) were inversely associated with the total distress experienced during the first 3 months (Figs. 1a and b). We have reported that members of the *Bifidobacterium* and *Lactobacillus* genus in intestinal microbiota appear to protect against crying and fussing and here we report that the specific *Bifidobacterium* species are associated with infant crying and distress. Identification of specific *Bifidobacterium* and *Lactobacillus* strains that would have optimal protective properties would benefit at-risk infants and result in new probiotic applications.

## Conclusion

Our findings link the composition of the gut microbiota to fussing and crying during early infancy in demonstrating species-specific effects of the gut microbiota on infant distress. A more profound understanding of the complex nature of infant crying is needed, as it is likely that there are distinct etiological factors and pathogenic mechanisms underlying the condition; detailed elucidation of these may facilitate the development of novel preventive and therapeutic options against this common problem.
